# Supplementary material for: Model of the Origin of a Ciguatoxic Grouper (Plectropomus leopardus)
Source: Toxins (Basel). 2023 Mar 21;15(3):230. doi: 10.3390/toxins15030230 (PMC10055633; doi:10.3390/toxins15030230)
Supplement: Supplementary file 1 [file toxins-15-00230-s001.zip › toxins-2220438-supplementary.pdf]

toxins

Table S1. Species of coral trout from the Great Barrier Reef, Australia [1].

Plectropomus spp.	Common/Local Name	*Variola* spp.	Common/Local Name
P. aereolatus	Passionfruit coral trout	*V. albimarginata*	White-edge coral trout
P. laevis	Blue-spotted coral trout	*V. louti*	Yellow-edge coronation trout
P. leopardus	Common coral trout		
P. maculatus	Bar-cheek coral trout		
P. oligacanthus	Vermicular cod		

[1] Fishes of Australia. Bray, D.J.; Gomon, M.F. Ed. Museums Victoria and OzFishNet, http://fishesofaustralia.net.au/ (last accessed on 4 March 2023).

Table S2. Total P-CTX burden in common coral trout (*P. leopardus*) with a muscle (flesh) concentration of 0.1 µg/kg of P-CTX-1 [1] based upon methodology of Holmes and Lewis [64].

Coral trout (kg)	Flesh (kg) based upon 50% recovery [2]	Flesh P-CTX-1 (µg) Burden at 0.1 µg/kg	Total P-CTX-1 (µg) Burden in Fish (40% of Toxin Burden)	Total P-CTX-1 (µg) Burden in Fish (10% of Toxin Burden)
1.6	0.8	0.08	0.2	0.8

[1] assuming flesh contributes 10–40% of total CTX burden by weight [88]. [2] [86,87].

Table S3. Estimated P-CTX burden in Great Barrier Reef trophic levels required to contaminate a 1.6 kg common coral trout muscle with 0.1 µg/kg P-CTX-1 based upon methodology of Holmes and Lewis [64].

Modelled Assimilation Efficiency for Each Trophic Level [1]	Trophic Level 3: Required P-CTX (µg) Burden in Common Coral Trout [2]	Trophic Level 2: Required P-CTX (µg) Burden in Surgeonfish	Trophic Level 1: Required P-CTX (µg) Load from *Gambierdiscus*	Cell Population of *Gambierdiscus* Producing 1.6 pg P-CTX-4B/cell
6%	0.2–0.8	3.3–13.3	55.6–222.2	34.8–138.9 × 10^6
43%	0.2–0.8	0.5–1.9	1.1–4.3	0.7–2.7 × 10^6
100%	0.2–0.8	0.2–0.8	0.2–0.8	0.1–0.5 × 10^6

[1] assimilation efficiency of 6.1% reported for P-CTX-1 in juvenile grouper [114] with a similar low-efficiency (<5%) for Caribbean-CTX in freshwater goldfish [123], 43% for Caribbean-CTX in pinfish [88] with a similar efficiency (42%) for CTX from *G. polynesiensis* in mullet [105], or 100% (no loss of toxin; for comparative purposes only). [2] assuming flesh contributes 10%–40% of total P-CTX burden by weight (data from Table S2).

Table S4. Conceptual model for the relative toxicity (arbitrary units) over 32 days of a school of surgeonfish with one fish from the school being eaten by a coral trout every fourth day (highlighted in yellow). The toxicity units are relative within each species, i.e., they are not comparable between species because losses and any potential toxin biotransformations are not incorporated. Under this scenario, a coral trout feeds on a fish from a school of surgeonfish that have accumulated toxicity from ingesting a short-lived bloom of ciguatoxic *Gambierdiscus* on turf algae, i.e., the relative toxicity of fish in the school of surgeonfish does not change over the 32 days. Data presented graphically in Figure 2a.

Day	Surgeonfish CTX burden (arbitrary units)	Coral trout CTX burden (arbitrary units)
0	1	0
1	1	1
2	1	1
3	1	1
4	1	1
5	1	2
6	1	2
7	1	2
8	1	2
9	1	3
10	1	3
11	1	3
12	1	3
13	1	4
14	1	4
15	1	4
16	1	4
17	1	5
18	1	5
19	1	5
20	1	5
21	1	6
22	1	6
23	1	6
24	1	6
25	1	7
26	1	7
27	1	7
28	1	7
29	1	8
30	1	8
31	1	8
32	1	8

Table S5. Conceptual model for the relative toxicity (arbitrary units) over 32 days of a school of surgeonfish with one fish from the school being eaten by a coral trout every fourth day (highlighted in yellow). The toxicity units are relative within each species, i.e., they are not comparable between species because losses and any potential toxin biotransformations are not incorporated. Under this scenario, a coral trout feeds on a fish from a school of surgeonfish that have accumulated toxicity from ingesting a persistent bloom of ciguatoxic *Gambierdiscus* on turf algae i.e., the relative toxicity of fish in the school of surgeonfish increases daily over the 32 days. Data presented graphically in Figure 2b.

Day	Surgeonfish CTX burden (relative, arbitrary units)	Coral trout CTX burden (relative, arbitrary units)
0	1	0
1	1	1
2	2	1
3	3	1
4	4	1
5	5	5
6	6	5
7	7	5
8	8	5
9	9	13
10	10	13
11	11	13
12	12	13
13	13	25
14	14	25
15	15	25
16	16	25
17	17	41
18	18	41
19	19	41
20	20	41
21	21	61
22	22	61
23	23	61
24	24	61
25	25	85
26	26	85
27	27	85
28	28	85
29	29	113
30	30	113
31	31	113
32	32	113

Tables S6–S8 describe the variables, values, and calculations used to model the transfer of CTX through a three trophic levels of a marine food chain on the GBR to produce a mildly toxic grouper.

Table S6. Model parameters for trophic level 3: *Plectropomus leopardus* (common coral trout) a predatory grouper.

Variable	Model values	Comments, assumptions, and calculations
Targeted P-CTX-1 concentration in flesh of grouper	0.1 μg/kg	0.1 μg/kg P-CTX-1 would likely cause mild poisoning in 2 out of 10 people [85]).
Total weight of grouper	1.6 kg	1.6 kg = average size of *P. leopardus* caught by the commercial fishery from the GBR [9]. This species can grow to >20 kg[1].
Flesh (fillet) recovery from grouper	50%	[86,87]. I.e, 0.8 kg flesh from a 1.6 kg *P. leopardus*.
CTX load in flesh of grouper	0.08 μg P-CTX-1	0.8 kg flesh weight · 0.1 μg/kg P-CTX-1 = 0.08 μg P-CTX-1.
Total CTX burden in grouper	0.2–0.8 μg P-CTX-1	Total CTX burden is estimated to be between 10%–40% of the flesh toxin concentration [64,88]. CTX burden based upon 10% flesh toxin concentration = 0.08 μg P-CTX-1 · 0.1 = 0.8 μg P-CTX-1. CTX burden based upon 40% flesh toxin concentration = 0.08 μg P-CTX-1 · 0.4 = 0.2 μg P-CTX-1

[1]Fishes of Australia. Bray, D.J.; Gomon, M.F. Ed. Museums Victoria and OzFishNet, http://fishesofaustralia.net.au/ (last accessed 4 March 2023).

Table S7. Model parameters for trophic level 2: Surgeonfish (*Ctenochaetus striatus*) grazing turf algae and then preyed upon by 1.6 kg grouper. We have based our model on a surgeonfish of optimal meal size for the grouper to simplify the modelling (4.6% of grouper's body weight, [89]). For a 1.6 kg coral trout this is assumed to be a 74 g surgeonfish, which is likely to have a total length between 16–17 cm, [12]).

Variable	Model values	Comments, assumptions, and calculations
The transfer rate for CTX between trophic level 2 and 3.	43%	Based upon an average net CTX assimilation of 43% in pinfish [88], also see Holmes and Lewis [64]. This term accounts for CTX losses between trophic levels. The actual transfer rates for the modelled species are not known.
C. striatus CTX load.	0.5–1.9 μg P-CTX-1 eq.	Based upon a 43% transfer rate. The surgeonfish was estimated to accumulate a CTX burden 2.3 times that of the modelled (targeted) load for the 1.6 kg *P. leopardus*. I.e., 2.3 times the 0.2—0.8 μg P-CTX-1 eq. range (Table S6) estimated for *P. leopardus* = total toxin burden for the surgeonfish of 0.5—1.9 μg P-CTX-1 eq. By considering the CTX load as P-CTX-1 eq., there is no assumption of where in the food chain the precursor toxins P-CTX-4A and -4B are converted to P-CTX-1.
Grazing rate for *C. striatus* on turf algae.	10 bites/min	Grazing rates will depend on many factors, including water temperature and sediment load within turf algae [63]. 10

		bites/min is a conservative estimate as higher feeding rates have been reported from French Polynesia [93]. However, feeding rates will likely vary throughout the day depending upon continuous interaction with the environment, including the risk of predation.
The time *C. striatus* spend grazing on turf algae each day.	9 h	*Ctenochaetus striatus* are diurnal feeders and 9 h is consistent with daily feeding time reported for this species in French Polynesia [93]. However, this will likely vary throughout the day and between seasons.
Bite area on turf algae by *C. striatus*.	0.7 cm^2	Estimated from the area of a grazing scar from the lower jaw of a ~18 cm fish (Figure 4c in Tebbett et al. [58]). This area ignores the bite area from the upper jaw, as Tebbett et al. [58] suggest it contributes minimally to biting. Bite size will likely vary with the gape of the mouth which will vary with fish size. The area grazed will also likely vary with the aspect and morphology of the turf algae surface.
The efficacy of the bite of a *C. striatus* to remove and ingest *Gambierdiscus* from turf algae.	50%	This rate is an assumption as there are no data available. The image of the grazing scar from an ~18 cm *C. striatus* shows spaces of various widths between the bristle-like teeth (Figure 4c in Tebbett et al. [58]). The ingestion of *Gambierdiscus* probably varies with the morphology of the species of turf algae being grazed and the amount of detritus within the turf algae layer (with the detritus being the target food for this surgeonfish). It is possible that during a bite, detritus blocks spaces between the teeth leading to more epiphytes being "combed" from the turfs.

Table S8. Model parameters for trophic level 1: *Gambierdiscus* epiphytic upon turf algae grazed by surgeonfish.

Variable	Model values	Comments, assumptions, and calculations
The transfer rate for CTX between trophic level 1 and 2.	43%	Based upon an average net CTX assimilation of 43% in pinfish [88], also see Holmes and Lewis [64]. This transfer efficiency is similar to that reported for CTX from *G. polynesiensis* to mullet (42%, [105]). The actual transfer rates for the modelled species are not known.

Total CTX load produced by a cell population of *Gambierdiscus* consumed by surgeonfish.	1.1–4.3 µg P-CTX-4A and/or -4B	Based upon a 43% transfer efficiency between trophic levels, i.e., 2.3 times 0.15–1.9 µg P-CTX-1 eq. (Table S7).
Gambierdiscus densities on turf algae.	0.1, 1, 10, 100, 1000 cells/cm^2	Hypothetical cell densities that span the range reported from 24 h benthic screen assays (Tester et al. [71] and references therein). We are not aware of any reports of cell densities ≥1,000 cells/cm^2.
Gambierdiscus CTX concentration (P-CTX-4B)	0.6 and 1.6 pg/cell	The analysis is mostly based upon 1.6 pg/cell P-CTX-4B, which we previously used to model production of a ciguateric Spanish mackerel [64]. 1.6 pg P-CTX-4B/cell is a hypothetical and very high CTX concentration. The highest known concentrations of P-CTX-4A and -4B were extracted from *G. polynesiensis* isolated from French Polynesia, with a maximum P-CTX-4B concentration of 0.4 pg/cell, 0.2 pg/cell of P-CTX-4A, and a combined maximum P-CTX-4A and -4B concentration from a single isolate of 0.6 pg/cell [32]. P-CTX-4A and -B are bio-transformed in the food chain to P-CTX-1.

Table S9. Minimum *Gambierdiscus* P-CTX-4A/B concentrations to produce a 1.6 kg coral trout with a flesh contamination of 0.1 μg/kg P-CTX-1. This scenario assumes surgeonfish grazing for 32 days on turf algae with *Gambierdiscus* densities of 1–1000 cells/cm^2.

***Gambierdiscus* density on turf algae (cells/cm^2)**	**Minimum *Gambierdiscus* P-CTX-4A/B concentration based upon the CTX flesh concentration of the grouper being 40% of the total CTX burden (pg/cell)**	**Minimum *Gambierdiscus* P-CTX-4A/B concentration based upon the CTX flesh concentration of the grouper being 10% of the total CTX burden (pg/cell)**
1	18.2	71.1
10	1.8	7.1
100	0.2	0.7
1000	0.02	0.07
